# Patients with SLE have higher risk of cardiovascular events and mortality in comparison with controls with the same levels of traditional risk factors and intima-media measures, which is related to accumulated disease damage and antiphospholipid syndrome: a case–control study over 10 years

**DOI:** 10.1136/lupus-2020-000454

**Published:** 2021-02-05

**Authors:** Sofia Ajeganova, Ingiäld Hafström, Johan Frostegård

**Affiliations:** 1 1Division of Gastroenterology and Rheumatology, Department of Medicine Huddinge, Karolinska Institutet, Stockholm, Sweden; 2 Department of Clinical Sciences, Rheumatology Division, Universitair Ziekenhuis Brussel, Vrije Universiteit Brussel, Brussels, Belgium; 3 Rheumatology Unit, Karolinska University Hospital, Stockholm, Sweden; 4 Section of Immunology and Chronic Disease, Institute of Environmental Medicine, Karolinska Institutet, Stockholm, Sweden

**Keywords:** antiphospholipid syndrome, lupus erythematosus, systemic, atherosclerosis, cardiovascular diseases

## Abstract

**Objective:**

SLE is a strong risk factor for premature cardiovascular (CV) disease and mortality. We investigated which factors could explain poor prognosis in SLE compared with controls.

**Methods:**

Patients with SLE and population controls without history of clinical CV events who performed carotid ultrasound examination were recruited for this study. The outcome was incident CV event and death. Event-free survival rates were compared using Kaplan-Meier curves. Relative HR (95% CI) was used to estimate risk of outcome.

**Results:**

Patients (n=99, 87% female), aged 47 (13) years and with a disease duration of 12 (9) years, had mild disease at inclusion, Systemic Lupus Erythematosus Diseases Activity Index score of 3 (1–6) and Systemic Lupus International Collaborating Clinics (SLICC) Damage Index score of 0 (0–1). The controls (n=109, 91% female) were 49 (12) years old. Baseline carotid intima-media thickness (cIMT) did not differ between the groups, but plaques were more prevalent in patients (p=0.068). During 10.1 (9.8-10.2) years, 12 patients and 4 controls reached the outcome (p=0.022). Compared with the controls, the risk of the adverse outcome in patients increased threefold to fourfold taking into account age, gender, history of smoking and diabetes, family history of CV, baseline body mass index, waist circumference, C reactive protein, total cholesterol, high-density lipoprotein, low-density lipoprotein, dyslipidaemia, cIMT and presence of carotid plaque. In patients, higher SLICC score and SLE-antiphospholipid syndrome (SLE-APS) were associated with increased risk of the adverse outcome, with respective HRs of 1.66 (95% CI 1.20 to 2.28) and 9.08 (95% CI 2.71 to 30.5), as was cIMT with an HR of 1.006 (95% CI 1.002 to 1.01). The combination of SLICC and SLE-APS with cIMT significantly improved prediction of the adverse outcome (p<0.001).

**Conclusion:**

In patients with mild SLE of more than 10 years duration, there is a threefold to fourfold increased risk of CV events and death compared with persons who do not have SLE with similar pattern of traditional CV risk factors, cIMT and presence of carotid plaque. SLICC, SLE-APS and subclinical atherosclerosis may indicate a group at risk of worse outcome in SLE.

**Key messages**

**What is already known about this subject?**

- Cardiovascular (CV) morbidity and all-cause mortality in SLE are increased compared with the general population.

**What does this study add?**

- In this analysis, the risk of CV events and death was threefold to fourfold higher in patients with SLE, even with mild disease, compared with persons who do not have SLE but with similar pattern of traditional CV risk factors and subclinical atherosclerosis measured with carotid intima-media thickness and presence of carotid plaque.
- The accumulated disease damage measured with the Systemic Lupus International Collaborating Clinics (SLICC) Damage Index and coexistence of SLE-antiphospholipid syndrome (SLE-APS) combined with measures of carotid intima-media thickness significantly improved prediction of the adverse outcome, beyond the prediction which was determined with these risk factors separately.

**How might this impact on clinical practice or future developments?**

- SLICC, SLE-APS and subclinical atherosclerosis may indicate a group at risk of worse outcome in SLE.
- This study provides data to support the need for a comprehensive approach to risk management in SLE, including assessment of both traditional risk factors, disease-specific characteristics and subclinical atherosclerosis.

## Introduction

SLE is a chronic systemic autoimmune disease that can involve any organ and is associated with significant morbidity and mortality. Despite marked improvement in 10-year survival of patients with SLE over the past five decades, mortality rates in SLE remain high compared with those in the general population.^1^ In the late 1940s, more than 40% of patients with SLE died within 3 years of onset of first symptoms.^2^ In the last decades, all-cause and cause-specific mortality ratio has significantly decreased over time, and the rate of 5-year and 10-year survival of patients with SLE has increased to greater than 90% due to improved treatment, earlier recognition of disease and management of comorbidities.^3 4^

Despite improvement in treatment and diagnosis, SLE is still one of the strongest known risk factors for cardiovascular (CV) events, even after controlling for traditional Framingham risk factors.^5 6^ Patients with SLE are suggested to have between 9-fold and 50-fold increased risk of developing CV events compared with the population of similar age without SLE, and this risk is remarkably increased in young women with SLE aged 36–45 years.^7 8^ SLE is also among the top 20 leading causes of death in females 5–64 years of age in 2000–2015.^9^ In patients with SLE particularly high mortality has been observed for circulatory disease, infections, malignancies and renal disease.^4 10^ Although difference in pathogenesis exists, the overlap between risk factors, which are historically classified as CV risk factors, has been suggested for both cardiovascular disease (CVD) and cancer development.^11 12^

We and others have shown a higher prevalence of atherosclerotic plaque within patients with SLE compared with controls, and associated risk factors have been described.^13 14^ However, there are only a few longitudinal studies reporting the significance of subclinical atherosclerosis for prognosis in SLE. It is suggested that prognosis in SLE is determined through an interplay between traditional CV risk factors, effects of SLE disease, antiphospholipid (aPL) antibodies, treatment-related effects and (subclinical) atherosclerosis, which together affect the outcome.^15–17^ However, it is not completely understood which factors, or conceivable combination of risk factors, are most important for premature CV morbidity and death in SLE. We took advantage of the earlier reported ultrasound case–control study with a follow-up of 10 years to investigate which factors could contribute to poorer prognosis in terms of CV events and mortality in SLE compared with controls.

## Patients and methods

### Patients and outcome assessment

Consecutive patients with SLE who fulfilled the 1982 revised criteria for SLE of the American College of Rheumatology^18^ and were younger than 70 years (n=118), and age-matched and sex-matched controls from the general population (recruited randomly from the same catchment area through the population registry) (n=122), were originally enrolled to the SLE Vascular Impact Cohort (SLEVIC) study. Patients with SLE were recruited from the outpatient clinic seen by rheumatologists at the Karolinska University Hospital; thus, the patient sample is representative of the tertiary care centre. The design of the original study has been described elsewhere.^13^ All participants were asked to perform carotid examination at inclusion and were followed longitudinally.

**Figure 1:**
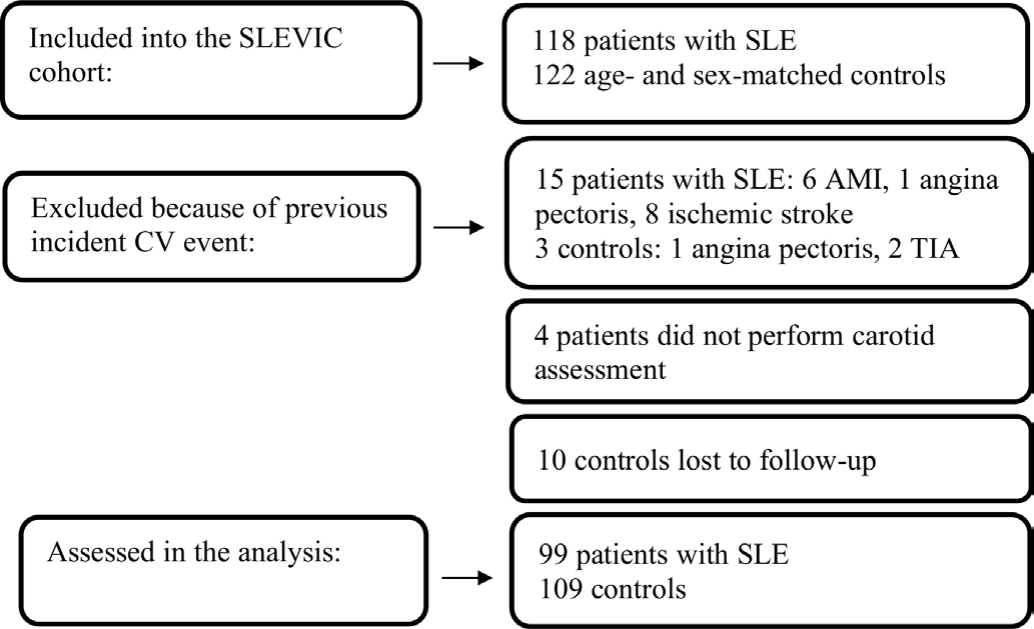
Study flow chart. AMI, acute myocardial infarction; CV, cardiovascular; SLEVIC, SLE Vascular Impact Cohort; TIA, transient ischaemic attack.

Information on CV events was obtained retrospectively and validated through a structured review of medical records. The CV events considered were acute myocardial infarction (AMI), hospitalisation for angina pectoris, coronary artery bypass grafting or percutaneous coronary intervention, ischaemic stroke, and transient ischaemic attack (TIA). Patients (n=15) and controls (n=3) with prevalent CV event prior to inclusion were excluded from the analysis; 4 patients missed carotid examinations and 10 controls were lost to follow-up (medical records unavailable). Therefore, the present study sample was composed of 99 patients and 109 controls (figure 1). The outcome of the study was the composite event of incident CV (ie, the first coronary artery or cerebrovascular CV event as defined above) or death from all causes.

The observation period started between September 2006 and January 2008, that is, when the patients and controls were included in SLEVIC and had carotid examinations. Participants were followed until the occurrence of the first-ever incident CV event, death or censoring date of March 2017, whichever occurred first. CV events and mortality data were recorded based on medical chart reviews. Causes of death were obtained from the death certificates.

The study participants provided written informed consent. It was not appropriate or possible to involve patients or the public in the design, or conduct or reporting plans of this study.

### Data collection

Structured data collection was performed at inclusion. All participants underwent a complete physical examination including blood pressure, body mass index (BMI), waist circumference measurement and submitted fasting blood samples. The following CV risk factors were registered: smoking history, hypertension (self-reported history of hypertension, hypertension by medical chart review, prescription of antihypertensive drugs for blood pressure-lowering or blood pressure ≥140/90 mm Hg at assessment for the study purpose), diabetes mellitus (history of diabetes mellitus, prescription of antidiabetic drugs or fasting blood glucose ≥7.0 mmol/L), dyslipidaemia (history of dyslipidaemia, medication prescription, low-density lipoprotein (LDL) >3.4 mmol/L or high-density lipoprotein (HDL) <1.0 mmol/L) and obesity (BMI ≥30 kg/m^2^).

For patients with SLE, aPL antibodies (lupus anticoagulant, anticardiolipin antibodies and β2-glycoprotein) were determined at the Karolinska Immuno Lab. The presence of repeated aPL antibodies, secondary antiphospholipid syndrome (APS) associated with SLE and history of nephritis (biopsy-proven class III–V) were recorded by chart review. APS was defined according to the Sidney criteria, defined by the occurrence of venous, arterial and/or small vessels thrombosis and/or pregnancy complications in the presence of aPL antibodies at moderate to high titres repeated at least 12 weeks apart.^19^ At the visit to the clinic SLE disease activity was assessed with the Systemic Lupus Erythematosus Diseases Activity Index (SLEDAI)^20^ without the immunological tests, and organ damage was measured using the Systemic Lupus International Collaborating Clinics (SLICC) Damage Index.^21^

### Carotid ultrasound

Carotid ultrasound was performed at inclusion as described in detail previously.^13 22^ The right and left carotid arteries were examined with a duplex scanner (Sequoia, Siemens Acuson, Mountain View, California, USA) using a 6 MHz linear array transducer. The far wall of the common carotid artery (CCA), 0.5–1.0 cm proximal to the beginning of the carotid bulb, was used for measurements of the carotid intima-media thickness (cIMT). cIMT was defined as the distance between the leading edge of the lumen-intima echo and the leading edge of the media-adventitia echo. Examinations were digitally stored for subsequent analyses by a computer system,^23^ with automated tracing of echo interfaces and measurements of distances between the wall echoes within a 10 mm long section of CCA in late diastole, defined by a simultaneous electrocardiographic recording. To exclude erroneous measures in case of cIMT thickening due to diffuse atherosclerotic plaque, the cIMT was not measured when a plaque was observed in the region of the CCA measurements. The mean values of the cIMT within the 10 mm long section were calculated. The mean cIMT (cIMT right+cIMT left)/2, was calculated. The difference between repeated measurements of cIMT was 4.9% (coefficient of variation) using the automated analysing system. Carotid plaque was defined as a localised intima-media thickening of >1 mm and at least 100% increase in thickness compared with adjacent wall segments. Plaque was screened for in the common, internal and external carotid arteries.

### Statistical methods

Descriptive statistics are reported as mean (SD) for continuous and percentages for categorical variables. To compare variables between the groups at baseline, t-test for equality of means and Mann-Whitney test for independent samples and χ^2^ and Fisher’s exact test were used as suitable.

The rates of event-free survival in patients and controls were compared using Kaplan-Meier analysis. Equality of time-to-event function between the groups was tested with log-rank test. Relative HRs from Cox proportional hazards regression models were used to estimate the effect of risk factors and carotid measurement on the composite outcome of incident CV events and death. To investigate if the combination of factors would improve prediction of the adverse outcome in patients, we used forward stepwise likelihood ratio (LR) method in the multivariate Cox proportional hazards regression analysis. Into these models we entered the variables which in univariate analyses were associated with the outcome at the level of significance of p<0.1.

Significance tests were two-tailed and conducted at the 0.05 level of significance. IBM SPSS V.26 was used for analyses.

## Results

At inclusion, the 99 patients with SLE were 47 (13) years, of whom 87% were women, and had a mean disease duration of 12 (9) years, median (IQR) SLEDAI (without the immunological tests) score of 3 (1–6) and SLICC score of 0 (0–1). The mean age of the 109 controls was 49 (12) years, of whom 91% were women (table 1).

**Table 1:** Baseline characteristics of patients with SLE and controls

	SLE, n=99	Controls, n=109	P value
Age, years	47.2 (13.2)	49.1 (12.1)	0.293
Female, %	86.9	90.8	0.363
CRP, mg/L	2.1 (0.8–5.2)	1.0 (0.5–2.4)	<0.001
ESR (mm/hour)	21.7 (15.8)	10.2 (8.1)	<0.001
Total cholesterol, mmol/L	4.7 (1.1)	4.8 (1.0)	0.402
LDL-cholesterol, mmol/L	2.5 (0.9)	2.8 (0.8)	0.055
HDL-cholesterol, mmol/L	1.6 (0.5)	1.7 (0.6)	0.234
Triglycerides, mmol/L	1.1 (0.6)	0.9 (0.4)	0.001
Smoking ever, %	53.5	54.1	0.932
Hypertension, %	52.5	24.8	<0.001
Diabetes mellitus, %	4.0	2.8	0.711
Dyslipidaemia, %	33.3	37.6	0.519
BMI, kg/m^2^	24.9 (4.6)	25.3 (4.5)	0.508
Family history of CV disease, %	23.2	35.8	0.048
Mean cIMT, µm	607 (127)	623 (118)	0.345
Carotid plaque, %	38.5	26.6	0.068

Values are expressed as mean (SD), median (IQR) or percentage as suitable.

P value represents comparison between the groups.

BMI, body mass index; cIMT, carotid intima-media thickness; CRP, C reactive protein; CV, cardiovascular; ESR, erythrocyte sedimentation rate; HDL, high-density lipoprotein; LDL, low-density lipoprotein.

Patients and controls were of similar age and did not differ by sex distribution, smoking habits, prevalence of diabetes mellitus, dyslipidaemia and BMI. Patients had higher levels of C reactive protein (CRP), erythrocyte sedimentation rate and triglycerides and were more likely to have hypertension, while the controls were more likely to report a family history of CV events (table 1).

### Incident CV events and death during follow-up in patients with SLE and controls

The overall median duration of follow-up was 10.1 (range 9.8–10.2) years. In all, 12 patients and 4 controls were registered with an incident CV event or died during follow-up. In patients, the cumulative incidence of such an outcome was 1.3 per 100 person-years at risk (95% CI for Poisson distribution, 0.56 to 2.02) and in the controls 0.4 per 100 person-years (95% CI 0.1 to 0.74). Among the patients, five cases experienced incident CV events (three cases of ischaemic stroke, two cases of TIA) and seven patients died (four cases due to cancer and three cases due to infections). According to the death certificates SLE was indicated as contributing to the deaths. Among the controls, two incident CV events (one case of AMI, one case of TIA) and two deaths (one cancer and one neurological disorder as the main cause of death) occurred during follow-up.

**Figure 2:**
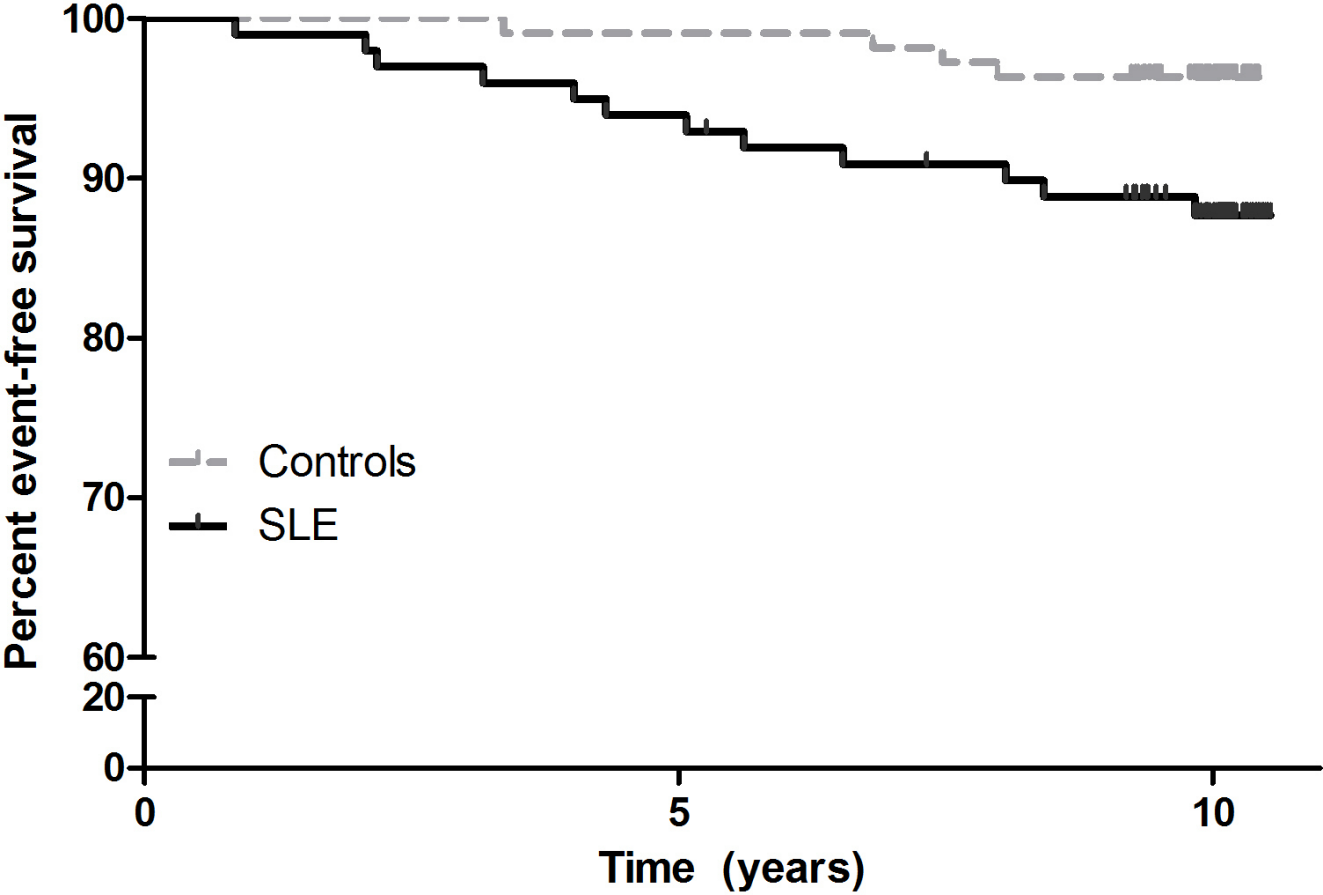
Kaplan-Meier estimates of the percentages of patients with SLE and controls without incident cardiovascular event and death during follow-up.

The adverse outcome occurred more often in patients than in the controls (p=0.022) by log-rank test (figure 2). The HR for the outcome was 3.7-fold (95% CI 1.2 to 11.5) higher in patients than in the controls, adjusted for age, sex and smoking history (p=0.025).

### Characteristics of participants by adverse outcome (incident CV events and death) during follow-up

#### Distribution of common CV risk factors

Patients with the adverse outcome during follow-up were older than those without (mean age 56 vs 46 years old, p=0.008) and more likely had higher cIMT (mean 714 µm vs 591 µm, p=0.008). Patients with the outcome had higher waist circumference and levels of triglycerides, more often had hypertension, diabetes mellitus and smoking history, were more likely to use antihypertensive and cholesterol-lowering medication, and less likely to use low-dose aspirin (although statistical significance was not reached). The controls with the adverse outcome during follow-up more often had diabetes mellitus, were more likely to use antihypertensive and cholesterol-lowering medication, and had a family history of CV events (table 2).

**Table 2:** Characteristics of patients with SLE and controls, grouped by the adverse outcome at follow-up

	SLE		Controls	
	Incident CV and death, n=12	Event-free, n=87	Incident CV and death, n=4	Event-free, n=105
Demographics at baseline				
Age, years	56.1 (12.7)**	46.0 (12.8)	50.2 (11.3)	49.0 (12.3)
Female gender, %	83.3	87.4	75.0	91.4
CV risk factors at baseline				
CRP, mg/L	3.9 (0.9–6.6)	1.9 (0.8–4.4)	1.1 (0.8–5.0)	1.0 (0.5–2.4)
ESR (mm/hour)	24.8 (21.6)	21.3 (14.9)	12.0 (10.2)	10.2 (8.0)
Total cholesterol, mmol/L	4.5 (1.3)	4.7 (1.1)	5.0 (0.4)	4.8 (1.0)
LDL-cholesterol, mmol/L	2.3 (0.9)	2.6 (0.9)	3.0 (0.2)	2.8 (0.8)
HDL-cholesterol, mmol/L	1.5 (0.3)	1.6 (0.5)	1.5 (0.5)	1.7 (0.6)
Triglycerides, mmol/L	1.5 (1.1)	1.0 (0.5)	1.0 (0.4)	0.8 (0.5)
Smoking ever, %	66.7	51.7	75.0	53.3
Hypertension, %	66.7	50.6	25.0	24.8
Diabetes mellitus, %	16.7	2.3	25.0	1.9
Dyslipidaemia, %	41.7	32.2	50.0	37.1
BMI, kg/m^2^	26.9 (6.2)	24.6 (4.3)	24.7 (2.4)	25.3 (4.6)
Waist circumference, cm	93.3 (13.1)	85.6 (12.3)	83.0 (9.0)	85.7 (12.0)
Family history of CV disease, %	33.3	21.8	50.0	35.2
Medication at baseline				
Antihypertensive, %	50.0	36.8	25.0	8.6
Cholesterol-lowering, %	16.7	6.9	25.0	4.8
ASA, %	8.3	19.5	0	1.9
Anticoagulants, %	16.7	5.7	0	1.0
Carotid measurements				
mean cIMT, µm	714 (153)**	591 (116)	673 (121)	621 (119)
Carotid plaque, %	41.7	38.1	25.0	26.7
SLE disease-specific characteristics			NA	
SLE duration, years	13.6 (7.2)	11.4 (9.6)		
SLEDAI	5.0 (2.0–7.8)	3.0 (1.0–5.3)		
SLICC	2.5 (1.0–3.0)**	0 (0–1.0)		
APS, %	54.5**	11.8		
aPL antibodies, %	50.0	36.8		
Lupus nephritis, %	44.4	43.4		
Current SLE medication				
DMARDs, %	66.7	44.2		
HCQ, %	41.7	50.6		
Prednisolone, %	75.0	59.8		
Prednisolone, mg/day	7.5 (5.0–10.0)	5.0 (2.7–7.5)		
SLE medication before inclusion				
Cyclophosphamide and/or rituximab, %	16.7	24.4		
HCQ, %	83.3	85.1		
Prednisolone cumulative dose, g	27.8 (11.2–39.0)	13.0 (5.4–24.2)		
Prednisolone cumulative duration, months	109.0 (59.0–178.8)*	54.8 (17.5–110.0)		
Prednisolone average dose, mg/day	5.6 (2.7–8.3)	4.5 (1.8–6.0)		

Values are expressed as mean (SD), median (IQR) or percentage as suitable.

P values for differences (with adverse events vs without them) are indicated as *p<0.05, **p≤0.01, ***p≤0.001.

Anticoagulants: coumarin derivatives and low molecular weight heparin; DMARDs: azathioprine, methotrexate, mycophenolate mofetil and ciclosporin.

aPL, antiphospholipid antibodies (lupus anticoagulant, anti-cardiolipine or anti-β2-glycoproteine); APS, antiphospholipid syndrome; ASA, low-dose aspirin; BMI, body mass index; cIMT, carotid intima-media thickness; CRP, C reactive protein; CV, cardiovascular; DMARDs, disease-modifying antirheumatic drugs; ESR, erythrocyte sedimentation rate; HCQ, hydroxychloroquine; HDL, high-density lipoprotein; LDL, low-density lipoprotein; NA, not applicable; SLEDAI, Systemic Lupus Erythematosus Diseases Activity Index (without the immunological tests); SLICC, Systemic Lupus International Collaborating Clinics Damage Index.

#### Distribution of SLE-specific risk factors

Patients with SLE and the adverse outcome during follow-up, in comparison with patients without, were more likely to have higher SLICC score (median 2.5 vs 0, p=0.002), history of SLE-APS (55% vs 12%, p=0.002), were exposed to a higher cumulative dose of prednisolone (median 28 g vs 13 g, p=0.056) and were treated with prednisolone (median 109 months vs 55 months, p=0.025) for a longer time. Patients with the outcome had longer disease duration, higher SLEDAI score and used anticoagulants more often (table 2).

### Risk factors of the adverse outcome

In patients with SLE, there was statistical evidence of shorter event-free survival at a higher age, higher levels of triglycerides and waist circumference, presence of diabetes mellitus, and higher cIMT, SLICC and SLE-APS (table 3). There was a trend for a poorer prognosis in case of higher BMI, higher cumulative dose and longer usage of prednisolone. After controlling for age and gender, the SLICC score and SLE-APS were still significantly associated with the adverse outcome: HR 1.66 (95% CI 1.20 to 2.28; p=0.002) and HR 9.08 (95% CI 2.71 to 30.5; p<0.001), respectively.

**Table 3:** HR for risk of incident CV events and death in patients with SLE and controls

	SLE		Controls		SLE vs controls	
Total patients, n	99		109		–	
Total events, n	12		4			

	HR (95% CI)	P value	HR (95% CI)	P value	HR (95% CI)	P value
Age	1.07 (1.01 to 1.13)	0.023	1.01 (0.93 to 1.09)	0.858	3.75 (1.21 to 11.6)	0.022*
Female gender	0.73 (0.16 to 3.34)	0.687	0.31 (0.03 to 2.96)	0.307	3.39 (1.09 to 10.5)	0.035*
CRP	1.02 (0.95 to 1.09)	0.674	1.06 (0.78 to 1.44)	0.711	3.28 (1.03 to 10.5)	0.045*
ESR	1.01 (0.98 to 1.05)	0.413	1.02 (0.93 to 1.12)	0.655	2.86 (0.85 to 9.6)	0.089
Total cholesterol	0.87 (0.51 to 1.46)	0.590	1.19 (0.45 to 3.15)	0.727	3.44 (1.11 to 10.7)	0.032*
LDL-cholesterol	0.71 (0.35 to 1.48)	0.363	1.45 (0.45 to 4.71)	0.537	3.36 (1.08 to 10.5)	0.037*
HDL-cholesterol	0.63 (0.18 to 2.29)	0.486	0.39 (0.03 to 4.97)	0.469	3.34 (1.08 to 10.4)	0.037*
Triglycerides	2.08 (1.14 to 3.80)	0.016	1.87 (0.30 to 11.6)	0.502	2.76 (0.86 to 8.82)	0.087
Smoking ever	1.80 (0.54 to 6.0)	0.336	2.61 (0.27 to 25.1)	0.406	3.5 (1.13 to 10.6)	0.030*
Hypertension	1.89 (0.57 to 6.29)	0.297	1.02 (0.11 to 9.8)	0.987	3.03 (0.94 to 9.7)	0.063
Diabetes mellitus	5.40 (1.18 to 24.7)	0.030	14.1 (1.46 to 137)	0.022	3.30 (1.06 to 10.2)	0.039*
Dyslipidaemia	1.43 (0.45 to 4.5)	0.544	1.67 (0.23 to 11.9)	0.608	3.53 (1.14 to 11.0)	0.029*
BMI	1.12 (0.99 to 1.27)	0.072	0.97 (0.77 to 1.22)	0.794	3.69 (1.18 to 11.5)	0.025*
Waist circumference	1.05 (1.01 to 1.09)	0.040	0.98 (0.90 to 1.07)	0.655	3.25 (1.05 to 10.1)	0.041*
Family history of CV disease	1.69 (0.51 to 5.61)	0.391	1.81 (0.26 to 12.9)	0.553	3.75 (1.20 to 11.7)	0.023*
Mean cIMT	1.006 (1.002 to 1.01)	0.003	1.003 (0.99 to 1.01)	0.389	4.05 (1.30 to 12.6)	0.016*
Carotid plaque	1.13 (0.36 to 3.55)	0.839	0.91 (0.09 to 8.67)	0.929	3.54 (1.13 to 11.04)	0.030*
SLE disease-specific risk factors						
SLE duration	1.03 (0.98 to 1.09)	0.301	NA			
SLEDAI	1.04 (0.93 to 1.17)	0.519				
SLICC	1.71 (1.28 to 2.29)	<0.001*				
APS	7.72 (2.35 to 25.4)	0.001*				
aPL antibodies	1.75 (0.56 to 5.43)	0.332				
Lupus nephritis	1.05 (0.28 to 3.9)	0.945				
Current SLE medication						
ASA	0.38 (0.05 to 2.96)	0.357				
Anticoagulants	3.21 (0.70 to 14.7)	0.133				
Prednisolone	1.97 (0.53 to 7.3)	0.308				
Prednisolone current dose	1.04 (0.91 to 1.19)	0.588				
SLE medication before inclusion						
DMARDs	2.46 (0.74 to 8.18)	0.141				
HCQ	0.87 (0.19 to 3.97)	0.858				
Cyclophosphamide and/or rituximab	0.62 (0.14 to 2.82)	0.534				
Prednisolone cumulative dose, g	1.02 (1.0 to 1.05)	0.071				
Prednisolone cumulative duration, year	1.07 (1.0 to 1.15)	0.059				
Prednisolone average dose, mg/day	1.09 (0.92 to 1.29)	0.316				

Values are HR for adverse events based on the Cox regression analysis, presented per each risk factor in the SLE group and controls separately.

HRs for adverse events in the SLE group were compared with HRs in the controls at the similar level of each of the estimated risk factors.

*P value significant after additional adjustment for age and sex.

Anticoagulants: coumarin derivatives and low molecular weight heparin; aPL: anti-phospholipid antibodies (lupus anticoagulant, anti- cardiolipine oranti-β2- glycoproteine)

aPL, antiphospholipid; APS, antiphospholipid syndrome; ASA, low-dose aspirin; BMI, body mass index; cIMT, carotid intima-media thickness; CRP, C reactive protein; CV, cardiovascular; DMARDs, azathioprine, methotrexate, mycphenolate mofetil, cyclosporin; DMARDs, disease-modifying antirheumatic drugs; ESR, erythrocyte sedimentation rate; HCQ, hydroxychloroquine; HDL, high-density lipoprotein; LDL, low-density lipoprotein; NA, not applicable; SLEDAI, Systemic Lupus Erythematosus Diseases Activity Index; SLICC, Systemic Lupus International Collaborating Clinics Damage Index.

Because the distribution of traditional risk factors in patients and controls was different, we further compared the risk of the outcome in the groups considering the differences in the prevalence of these factors. The grouping variable (patients vs controls) and each of the risk factors were entered into Cox regression models; in this way, the risk of outcome was estimated at the similar level of the traditional risk factors. In these analyses we found that the risk of the adverse outcome was increased at the rate of three to four times in patients compared with controls at a similar level of such factors as age, gender, baseline levels of CRP, total cholesterol, HDL, LDL, smoking, diabetes mellitus, dyslipidaemia, BMI, waist circumference and carotid measures of intima-media thickness and plaque (table 3). The association of these factors with the outcome was still stronger in patients in comparison with controls after additional adjustment for age and gender (table 3).

Disease duration, SLEDAI at inclusion, history of lupus nephritis, treatment with anticoagulants, traditional disease-modifying antirheumatic drugs, hydroxychloroquine, cyclophosphamide and rituximab were not associated with the adverse outcome in this study.

#### Combination of risk factors with cIMT to predict the adverse outcome

We further examined whether a combination of factors would improve prediction of the adverse outcome in patients. The addition of cIMT to SLICC statistically significantly improved the fit of the regression model (p<0.001), that is, the model including these two variables together predicted the outcome better than the models based on the SLICC score or the cIMT measure separately. In the model including both cIMT and SLICC, the HR (95% CI) for the outcome was 1.005 (1.001 to 1.01; p=0.016) for the effect of cIMT and 1.58 (1.18 to 2.13; p=0.002) for the SLICC score. Similarly, addition of cIMT to SLE-APS significantly improved the prediction of the outcome (p<0.001): HR (95% CI) 1.007 (1.003 to 1.011; p<0.001) for cIMT and 9.14 (2.71 to 30.8; p<0.001) for SLE-APS. The variables cumulative prednisolone use, triglycerides, diabetes mellitus, BMI and waist circumference were rejected as these were not significant from the forward stepwise LR analyses.

## Discussion

In this case–control study with a follow-up of 10 years we confirmed an excess of incident CV events and death in a cohort of patients with mild SLE disease of more than 10 years duration in comparison with persons of similar age and sex who did not have SLE. We showed that the risk of the adverse outcome was three to four times higher in patients with SLE compared with the controls considering the level of traditional CV risk factors, carotid measurements of cIMT and carotid plaque. Patients with the adverse outcome had higher SLICC score, cIMT and SLE-APS and had used prednisolone for a longer time compared with patients without the outcome. The combination of SLICC score and APS with the carotid measure of cIMT improved the prediction of the outcome. This suggests that accumulated disease damage, SLE-APS and subclinical atherosclerosis may indicate a group at risk of worse prognosis in patients with SLE.

In our case–control study we confirm that patients with established SLE still develop important long-term adverse events at a higher rate than population controls. This finding is in line with other studies reporting that although SLE mortality rates and the ratios of SLE mortality rates relative to non-SLE mortality rates have decreased every year since the late 1990s, they remain disproportionally high, and mortality in SLE is persistently higher than that in the general population.^1 24^

Earlier, an excess mortality in SLE was supposed to follow a bimodal pattern, with the early peak predominantly attributable to active lupus and its complications, and the later peak largely attributable to atherosclerosis and traditional CV risk factors.^15 25 26^ In our cohort of patients with SLE with mild SLE disease of more than 10 years duration, both traditional risk factors (age, triglycerides, waist circumference, diabetes mellitus) and disease factors (SLICC disease damage score and SLE-APS) and measures of subclinical atherosclerosis (cIMT) were associated with poor prognosis. Our observation is in line with long-term follow-up studies which revisited the concept of the bimodal pattern of mortality in SLE and have shown the importance of both disease severity and moderate to severe atherosclerosis for death of any cause in patients with SLE after a prolonged duration of the disease.^27^

In this study we took advantage of the direct comparison between patients with SLE and controls without SLE, in whom CV risk factors and carotid measures were assessed following the protocol of the study. We found that patients with SLE had a threefold to fourfold significantly higher risk of poor prognosis adjusted for demographic characteristics (age and gender), baseline levels of systemic inflammatory measure (CRP) and traditional CV risk factors (baseline total cholesterol, HDL, LDL, smoking, diabetes mellitus, dyslipidaemia, BMI and waist circumference) and subclinical atherosclerosis measures (cIMT and carotid plaque). These findings are in agreement with the evidence that common CV risk prediction algorithms recommended in the general population have low specificity in the context of an autoimmune disease and underestimate actual CV risk in an individual patient with SLE.^5 6 28^ However, traditional CV risk factors in patients with SLE should not be underestimated. Distinct CV risk factors separate patients with SLE with manifest CVD from patients with SLE without CVD and from population controls.^29^ The prevalence of CV risk factors in SLE is increased and they are associated with poor prognosis.^30 31^ Regular monitoring for traditional risk factors and management of traditional risk factors, in particular modifiable ones, are recommended as part of the disease management strategy to improve long-term outcomes in SLE.^32^ In the multinational, multiethnic inception cohort of patients with SLE followed up yearly between 1999 and 2017, it has been recently demonstrated that the prevalence of atherosclerotic vascular events accrual in the current era is much lower than that seen in previously published data: 3.6% vs 10% of patients.^33^ This decline in CV events may be attributable to better control of lupus disease activity, more judicious use of glucocorticoids, as well as improvements in the treatment of classic cardiac risk factors in the modern era.^34–36^

Detection of subclinical atherosclerosis long before clinical manifestations of atherosclerotic vascular events is important in identifying the risk group for future adverse events. Existing subclinical atherosclerosis, not identified through traditional CV risk factors or scores, has been demonstrated in patients with SLE using a variety of techniques including carotid ultrasound.^37^ It has been reported that patients with SLE have a relative risk of subclinical atherosclerosis, at least, comparable with patients with diabetes mellitus, a protopic disease for CV risk.^38^

Notably, patients with SLE in our study had baseline cIMT measures which were not statistically different from those in the control population; however, the incidence of CV events and death in patients was higher than in the controls, and baseline cIMT was associated with risk of poor prognosis in patients. In contrast to the here observed similar cIMT in patients and controls, one meta-analysis has concluded that patients with SLE, compared with healthy individuals, have a significantly increased cIMT of 0.08 mm (95% CI 0.06 to 0.09), but the included studies were markedly heterogenous.^39^ The variation in estimated cIMT is presumably related to different SLE population settings, particularly age at inclusion, duration of disease, extent of disease-induced damage, treatments, prevalence of CV risk factors and established atherosclerosis. It is important to point out that a previous study has found a greater cIMT in patients with SLE with CVD than in patients with SLE without CVD and population controls. Moreover, cIMT in patients with SLE without CVD was not different from that in population controls.^29^ The selection of patients without previous CV events for the present analysis is likely to explain the similar cIMT findings in patients and controls. Thus, patients with previous CV events in our original cohort (who were excluded from this analysis) had a greater cIMT than those without clinical CVD (included in this analysis): mean (SD) 696 (135) µm vs 607 (127) µm (p=0.009). A previous case–control study in patients with lupus with disease characteristics similar to those in our study has reported a significantly lower cIMT in patients than in controls who had a higher blood pressure than the patients,^14^ which also exemplifies the known influence of CV risk factors on the variation in intima-media thickness measures in different clinical settings.

The overall reported prevalence of carotid plaque was higher among patients with SLE than among the controls, especially the youngest age group.^14^ Also in our original SLEVIC cohort, which is the base for the present study, the prevalence of atherosclerotic plaques and also echolucent potentially vulnerable plaques was significantly increased among patients with SLE as compared with controls.^13^ The exclusion of participants with previous CV events for this study (more patients than controls, 15 patients vs 3 controls), as well as a higher prevalence of carotid plaque in the present controls compared with other reported controls (26.6% vs 15%–17%^40 41^), together with the small numbers in each group, likely precluded the difference in carotid plaque prevalence between patients and controls from being statistically significant (p=0.068). The here observed prevalence of carotid plaque in patients was similar to that in other observational studies of SLE (about 40%).^41 42^

Faster progression of carotid atherosclerosis in patients compared with the general population could explain an accelerated atherosclerotic disease process. We have, however, shown similar cIMT progression over 7 years in patients with mild SLE and the controls.^43^ Carotid plaques could be a stronger marker of atherosclerotic vessel disease and a predictor of clinical events, when compared with cIMT. Interestingly, the plaque progression rate was higher than, and the intima-media thickness progression rate was similar to, those in the control group in the Pittsburgh lupus cohort.^41^ Significantly higher OR of plaque progression in SLE compared with controls has also been found in the 3-year follow-up carotid and femoral artery ultrasound study,^44^ but the significance of this finding for the clinical outcome was not reported. Other studies have found that baseline carotid ultrasound measures in SLE, neither cIMT nor presence of carotid plaque, could accurately predict future CV events.^45^ Altogether, these observations suggest that the definition of premature subclinical atherosclerosis associated with clinically important long-term outcomes may differ in SLE and the general population, particularly in the presence of those factors augmenting the risk of poor prognosis. Further, quantitative ultrasound technique measuring total plaque burden and qualifying plaque could be a more accurate surrogate measure of future CV event. Thus, it has been suggested that the atherosclerotic plaques in SLE could be more vulnerable,^13^ a feature that is associated with a risk of occlusive events irrespective of the size of the plaque. Also, an ultrasound study in SLE from Toronto has shown that plaque area is a more accurate surrogate measure of future CV event and is more strongly associated with coronary artery disease and CV risk factors than cIMT.^46^

Patients with the adverse outcome in our study, compared with patients without, were more likely positive for aPL antibodies (50% vs 36.8%), have SLE-APS (54.5% vs 11.8%) and more frequently used anticoagulants (16.7% vs 5.7%). Of importance, all five observed CV events during follow-up in our patients were due to cerebrovascular events. Of these, three patients were triple positive for aPL antibodies and were diagnosed with SLE-APS, two more patients were not diagnosed with APS, but one of them was double positive for aPL antibodies (lupus anticoagulant and anti-β2-glycoprotein). However, only one of four patients who died with cancer as the main cause of death was triple positive for aPL antibodies and had SLE-APS; furthermore, one of three patients who died due to serious infection had SLE-APS positive for lupus anticoagulant, while other patients with these outcomes did not have aPL antibodies.

These findings are in line with prospective data showing significant morbidity and mortality in patients with APS despite current treatment.^47 48^ Although treatment of traditional CV risk factors according to current guidelines for the prevention of CVD in the general population is recommended for primary prevention in APS, there is a need for additional ways to assess and identify patients with SLE with potentially higher risk of poor prognosis. Because specific pathways are suggested in SLE-APS, such as endothelial dysfunction, accelerated endothelial proliferation and intimal hyperplasia, atherogenesis, platelet activation and coagulation-fibrinolytic dysregulation along with accelerated endothelial proliferation, intimal hyperplasia and early subclinical atherogenesis, stratification of risk factors, including SLE-specific and potentially antibody-specific risk stratification algorithms, could be proposed. Noteworthy, not all individuals with aPL develop thrombotic complications during their lifetime. The prognostic value of aPL positivity in SLE could be over and above that which can be related to thrombotic events. In SLE with end-stage renal disease, patients with aPL had higher risk of all-cause mortality than patients without these antibodies, while there was no association between presence of aPL and mortality in non-SLE patients with end-stage renal disease.^49^ In an early SLE study, causes of death in the aPL-positive group were cancer, infection and vascular events, although in the aPL-negative group infection and vascular-related events were also observed as the most frequent causes of death.^50^ These observations suggest that the presence of aPL, even in individuals without thromboses, could be associated with poor prognosis.

Accumulated disease damage measured with SLICC and SLE-APS were associated with increased risk of the adverse outcome during follow-up in our study. Previous studies have also highlighted important prognostic information of accumulated disease burden and coexistence of SLE-APS and aPL antibodies for CV outcomes in SLE.^51–55^ Moreover, we here show that considering the SLICC score, SLE-APS and cIMT measure together improved the prediction of risk of poor outcomes in SLE, beyond the prediction which was determined with these risk factors separately. This finding supports the view of the interplay of multifactorial pathways of atherosclerosis in SLE and suggests a need for a systematic individualised comprehensive risk assessment which also includes assessment of specific disease characteristics such as SLE-APS.

Our data broaden the evidence base for the harmful long-term effects of prolonged glucocorticoid use in SLE. We found that patients with SLE exposed to prednisolone for a longer time had a significantly higher likelihood of developing CV events or were deceased during follow-up compared patients who used prednisolone for a shorter period. Also, there was an indication that both cumulative time spent on prednisolone treatment and the cumulative dose of prednisolone used could be associated with poorer long-term prognosis. However, because of the limited number of patients, these analyses may not generate powerful statistically significant results. In a recent study, both the dose and intensity of longitudinal use of glucocorticoid were associated with cumulative burden of cardiovascular, musculoskeletal, gastrointestinal, ophthalmological, infectious, neuropsychiatric, metabolic and dermatological adverse events.^56^ This points to the need for optimised handling of glucocorticoid regimens both in order to first sufficiently suppress disease activity and thus to avoid or limit the disease damage, and second to minimise adverse events due to prolonged use of glucocorticoid in high doses.

A possible explanation for the excess of cerebrovascular events but not ischaemic coronary events among our patients could be that a good part of the included patients were middle-aged or older and had a long-lasting disease, although an increased risk of myocardial infarction is reported to be particularly apparent in young adults with SLE, with majority of women aged less than 55 years at the time of their first cardiac event.^8^ Another explanation could be that some pathways, for example, presence of APS, could be more involved in the occurrence of cerebrovascular events than coronary events. Alternatively, the here observed excess of cerebrovascular events could occur by chance due to the small sample size.

The main strengths of our study are the direct comparison of patients with SLE and population controls without SLE, assessment of all traditional CV risk factors and carotid measurements in all participants according to the protocol of the study, and a long follow-up of over 10 years sufficient to study morbidity and mortality. We also acknowledge the limitations of a relatively limited number of patients and missing follow-ups in some controls. This investigation focused on CCA intima-media thickness and quantification of plaque burden was unavailable. Although we could not exclude contribution of variation in disease activity during follow-up to the outcome, we examined the cumulative effects of disease severity, at least indirectly, through the SLICC damage score and the cumulative exposure to the prednisolone at inclusion. Because of the small number of patients in the analysis, the stratification by aPL antibodies profile was not possible and relevant effects of some risk factors may not have been detected. We used here the composite outcome of CV events and all-cause mortality, which is likely a complete assessment of the clinical prognosis. We recognise that competing risk of death was possible in some, if not all, of our patients who died due to cancer and infections and thence who may not have lived long enough to occur with the CV outcome of interest. Because the deaths among the patients were due to cerebrovascular events, it is unclear if the observed associations could also transfer to ischaemic coronary events. Nevertheless, in this study even mild SLE indicated a worse prognosis compared with controls, which was related to a variety of risk factors. These risk factors included those on, or requiring, SLE-specific treatments and some traditional CV risk factors, which are important findings.

## Conclusion

In this study, we show that taking into account the level of traditional CV risk factors, the long-term risk of incident CV events and all-cause death in patients with SLE, even with mild disease, is increased compared with population controls. For better management of CV risk and early mortality in patients with SLE, there is a need for comprehensive assessment including the following factors, in addition to traditional CV risk factors: accumulated disease damage, specific disease characteristics such as SLE-APS and subclinical atherosclerosis.

## Data Availability

All data relevant to the study are included in the article.
